# Pharmacological Efficacy of Repurposing Drugs in the Treatment of Prostate Cancer

**DOI:** 10.3390/ijms24044154

**Published:** 2023-02-19

**Authors:** Tânia Lourenço, Nuno Vale

**Affiliations:** 1 OncoPharma Research Group, Center for Health Technology and Services Research (CINTESIS), Rua Doutor Plácido da Costa, 4200-450 Porto, Portugal; 2 Faculty of Pharmacy, University of Porto, Rua de Jorge Viterbo Ferreira, 228, 4050-313 Porto, Portugal; 3 CINTESIS@RISE, Faculty of Medicine, University of Porto, Alameda Professor Hernâni Monteiro, 4200-319 Porto, Portugal; 4 Department of Community Medicine, Information and Health Decision Sciences (MEDCIDS), Faculty of Medicine, University of Porto, Rua Doutor Plácido da Costa, 4200-450 Porto, Portugal

**Keywords:** prostate cancer, drug repurposing, non-cancer drugs, approved drugs

## Abstract

Worldwide, prostate cancer (PC) is the second most frequent cancer among men and the fifth leading cause of death; moreover, standard treatments for PC have several issues, such as side effects and mechanisms of resistance. Thus, it is urgent to find drugs that can fill these gaps, and instead of developing new molecules requiring high financial and time investments, it would be useful to select non-cancer approved drugs that have mechanisms of action that could help in PC treatment, a process known as repurposing drugs. In this review article, drugs that have potential pharmacological efficacy are compiled to be repurposed for PC treatment. Thus, these drugs will be presented in the form of pharmacotherapeutic groups, such as antidyslipidemic drugs, antidiabetic drugs, antiparasitic drugs, antiarrhythmic drugs, anti-inflammatory drugs, antibacterial drugs, antiviral drugs, antidepressant drugs, antihypertensive drugs, antifungal drugs, immunosuppressant drugs, antipsychotic drugs, antiepileptic and anticonvulsant drugs, bisphosphonates and drugs for alcoholism, among others, and we will discuss their mechanisms of action in PC treatment.

## 1. Introduction

Worldwide, prostate cancer (PC) is the second most frequent cancer among men and the fifth leading cause of death [1]. In the initial stage, PC can be asymptomatic or manifest itself through difficulty with urination and frequent urination, as well as nocturia. In more advanced cases, symptoms include urinary retention and, in cases of bone metastasis, back pain may occur [1].

**Scheme 1:**
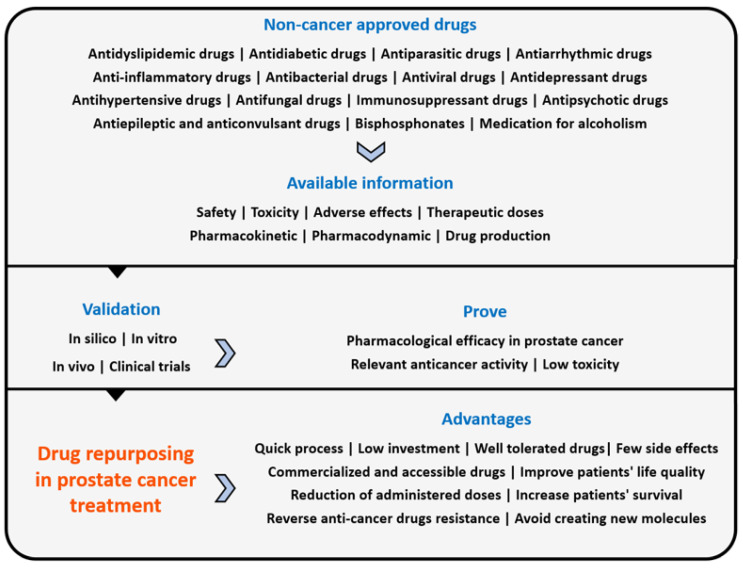
Repurposing drugs process in prostate cancer and its advantages. Non-cancer approved drugs already have available data and information that are useful for drug repurposing. After the selection of potential drugs, it is necessary to validate their properties to prove that they have what is needed to be used in prostate cancer treatment.

In recent years, the concept of repurposed drugs has emerged, and it consists of identifying and developing new therapeutic indications for existing drugs [2,3,4]. Repurposing drugs has many advantages (Scheme 1), because these drugs have already been comprehensively studied and there is already a lot of information available about them, such as their safety, toxicity and side effects, therapeutic doses, pharmacokinetic and pharmacodynamic properties and drug production, which reduces investigation time and financial investments in pre-clinical trials and phase I and II clinical trials. The literature also reports that most repurposed drugs are well tolerated by oral administration, and as many of these drugs are already accessible on the generic drug market, they have become available to all people [5,6].

Therefore, the number of repurposed drugs for several diseases has increased, just like in oncology, an area in which there is a particular interest in this topic, not only to prevent the significant side effects that are associated with chemotherapy, and which have high impact on cancer patients’ life quality, but also to increase patients’ survival and reverse resistance to antineoplastic drugs. Therefore, the objective of repurposing drugs in oncology is related to the identification of drugs whose original approval is not antineoplastic, but that present anticancer activity, taking into account that these drugs must not have significant side effects [2,4,6].

On the other hand, the literature mentions that the use of combined pharmacological therapies of two or three drugs with different mechanisms of action and signaling pathways can be useful, because they trigger a synergistic response that reduces the concentrations of individual drugs to optimize the therapy, increase the success rate and minimize/delay drug resistance. So, in combined therapy, a chemotherapeutic drug can be used with a repurposed drug, and particularly in PC, several drugs seem to be potential candidates for drug repurposing and combination therapy, with the advantage of being able to be used in other cancer types, because different tumors may have the same signaling pathways [2,7,8]. This study is extremely important, because it presents drugs organized by pharmacotherapeutic class that can be repurposed for PC treatment, distinguishing itself from other works by presenting not only drugs that are widely discussed in other literature reviews but also drugs that are very promising but less discussed, such as saquinavir, which shows good results when combined with conventional chemotherapy. Therefore, this review can open new doors in oncological treatments, supporting the repurposing of drugs instead of developing new molecules.

## 2. Pharmacotherapeutic Groups with Potential Efficacy in PC Treatment

### 2.1. Antidyslipidemic Drugs

In general, the severity of PC depends on the levels of circulating androgens, mainly testosterone, which, like other androgens, is a cholesterol derivative [9]. Thus, patients with PC generally have high cholesterol production because PC cells depend more on the endogenous production of cholesterol than dietary cholesterol. For that reason, men with high levels of serum cholesterol are at greater risk of developing PC, and it is known that cholesterol also plays an important role in the growth, proliferation, migration and invasion of cancer cells [10].

So, it is known that androgen deprivation therapy, the standard treatment for PC, inhibits androgen synthesis in testicles and, consequently, decreases the levels of systemic androgens, but despite the low availability of circulating androgens, tumor progression continues, because there is an intratumoral synthesis of androgens from the existing cholesterol [11]. Thus, statins are antidyslipidemic drugs that inhibit 3-hydroxy-3-methylglutaryl coenzyme A (HMG-CoA) reductase and block mevalonate production, which is a cholesterol precursor that allows for controlling the synthesis of androgens from cholesterol, which could explain the synergistic effect of statins in androgen deprivation therapy [2,5,10,11]. On the other hand, it is known that the metabolic pathway of mevalonate is associated with Yes-Associated Protein (YAP) and Tafazzin protein (TAZ), which are related to tumor growth and tumor progression. Thus, statins inhibit HMG-CoA reductase, blocking mevalonate production and inhibiting YAP/TAZ [12].

Therefore, statins reduce serum cholesterol levels, and in vitro and in vivo studies indicate that they reduce PC progression and the incidence of lethal PC, improve prognosis and survival rates and decrease the tumor volume and serum levels of the Prostate-Specific Antigen (PSA), and they are also radiosensitizing agents (Table 1). Radiotherapy is one of several standard PC treatments [2,5,10,12]. In addition to this, statins have demonstrated antiproliferative, anti-inflammatory and antioxidant properties, antitumor effects, an ability to suppress the production of essential metabolites for different cellular functions and for post-translational modification of cell signaling proteins and the capability to inhibit angiogenesis, metastases and tumor proliferation [12].

It is also known that statins reduce low-density lipoprotein (LDL) (30 to 60%), total cholesterol (23 to 28%) and triglycerides (25 to 45%), which is extremely useful in PC treatment, since in this tumor, there is a deregulation of lipid metabolism that results in excessive lipid accumulation. Thus, statins will eventually act on both serum cholesterol and intratumoral cholesterol, which is an advantage, because men with high serum cholesterol seem to have a higher risk of developing more aggressive PC and higher mortality rates. On the other hand, significant amounts of statins were detected in the prostate tissue of several patients, which allows us to conclude that statins have a direct effect on prostate cells, and after a four-week treatment with statins, it is already possible to detect them in prostatic tissue [11].

Some of the most important statins that can be repurposed for PC treatment are atorvastatin, fluvastatin, simvastatin mevastatin, lovastatin and rosuvastatin, which can be administered in monotherapy or in combination with other drugs, although recent studies indicate that the main focus is currently on atorvastatin and simvastatin [12]. In a clinical trial with men with advanced PC, atorvastatin reduced serum PSA levels when compared to the placebo and after 27 days of administration, it decreased the tumor proliferation. In another study, after the administration of 80 mg of atorvastatin for 27 days before prostatectomy, it was possible to detect the drug in the prostatic tissue of 50% of the evaluated men. On the other hand, atorvastatin also presents benefits when combined with radiotherapy, since it increases the radiosensitivity of hypoxia-induced PC cells by decreasing the HIF-1α expression. Other studies have shown that when prostatectomy is preceded by the administration of fluvastatin, significant apoptosis occurs, and after 4 to 12 weeks of the administration of 80 mg of fluvastatin, this drug was detected in the prostate tissue of 10 of the 28 patients evaluated with localized PC. Moreover, dipyridamole, an antiplatelet agent, when administered in combination with fluvastatin, reduces the amount of fluvastatin needed to induce the death of PC cells. Concerning simvastatin, it is known that it can resensitize PC cell lines that have developed resistance to enzalutamide (a second-generation non-steroidal antiandrogen that competes with androgens for binding sites in the prostate) and that acts through apoptosis caused by cholesterol depletion and inhibition of the Protein Kinase B (Akt) signaling pathway [5,10,11,12,13,14]. On the other hand, it also prevents tumor growth, reduces PSA levels and, when administered in combination with irinotecan, suppression of tumor growth and induction of apoptosis caused by inhibition of the MCL-1 protein occur [12].

**Table 1:** Examples of radiosensitizer drugs that enhance the lethal effects of radiation and have anticancer properties.

Drug Class	Drug	Anticancer Mechanism	References
Antidyslipidemic drugs	Statins	HMG-CoA reductase inhibitor; block mevalonate pathway	[2,5,10]
Anti-inflammatory drugs	Diclofenac	COX inhibitor; prostaglandin inhibitor; MYC inhibitor; regulates glucose metabolism	[2]
Antiviral drugs	Nelfinavir	S2P activity inhibitor; inhibits regulated intramembrane proteolysis of sterol regulatory element binding protein-1; Akt inhibitor; blocks androgen receptor signaling; down-regulates the androgen receptor	[2,5,15]
	Saquinavir	Inhibition of the 20s and 26s proteasome; NF-κB inactivator; PI3K-Akt inhibitor	[16]
Antiparasitic drugs	Chloroquine/Hydroxychloroquine	Interaction with nucleotides; apoptosis induction; autophagy inhibitor; elimination of cancer stem cells	[5,17]
Other drugs	Naftopidil	Akt phosphorylation inhibitor	[2,5]

Regarding lovastatin and simvastatin, they inhibit the activity of RhoA protein, activate caspase-3, caspase-8 and caspase-9, induce apoptosis, reduce the expression of Retinoblastoma Protein (Rb), phosphorylate Rb, D1 and D3 cyclins and CDK4 and CDK6, and increase the expression of p21 and p27 in PC cells [12]. Atorvastatin, mevastatin, simvastatin and rosuvastatin decrease the colonies of PC cells and reduce their migration due to the inhibition of Geranylgeranyl Pyrophosphate (GGPP) synthesis [12]. Furthermore, a recent study showed that statins, particularly fluvastatin, pravastatin, rosuvastatin, atorvastatin, cerivastatin, simvastatin and lovastatin, when administered at a high cumulative defined daily dose (CDDD), were associated with a lower risk of PC, and its use for more than five years can reduce the incidence of PC, which leads to the conclusion that high and long-term DDCDs are associated with a lower risk and lower incidence of PC and that they can be used to prevent its onset [18].

Additionally, despite not belonging to the statin group, fenofibrate is also an antidyslipidemic drug that activates Peroxisome-Proliferator-Activated Receptor α (PPARα), which plays a key role in lipid metabolism, having the ability to increase lipolysis. Therefore, at the PC level, fenofibrate induces apoptosis in tumor cells, reduces their dissemination and complicates the metastasis process due to cyclin D1 and E2F1 protein inhibition and the increase in the pro-apoptotic protein Bax [12,19]. Fenofibrate also decreases androgen receptors’ expression and their target genes by decreasing the levels of phosphorylated Akt and induces apoptosis by producing oxidative stress, including Superoxide Dismutase (SOD) inhibition [12].

So, both statins and fenofibrate have antiproliferative, antimetastatic and apoptotic properties in PC cells and also in other types of cancers, such as melanomas, neuroblastomas and gastric and hepatic cancer, among others [2,19].

### 2.2. Antidiabetic Drugs

Type 2 diabetes mellitus represents about 90% of diabetes cases and is one of the risk factors for the development of several cancers, such as liver, pancreas, colorectal, kidney, bladder, endometrium and breast cancer. In addition, diabetes is also associated with high levels of mortality in oncological situations, namely in PC, but several studies have shown that diabetes mellitus seems to reduce the risk of PC incidence, which appears to be related to the low plasma levels of Insulin-Like Growth Factor 1 (IGF-1) in diabetic people compared to in non-diabetic people [20].

Metformin is an antidiabetic drug that belongs to the biguanide group and decreases insulin tissue resistance and hepatic glucose production and, consequently, reduces serum glucose levels. In addition, metformin has also shown good results in PC treatment because it promotes apoptosis in PC cells since it deregulates the Phosphoinositide 3-Kinase (PI3K) signaling pathway and minimizes growth, proliferation and tumor cell differentiation. Metformin is also associated with a reduction in the incidence and mortality of PC, showing good results when combined with chemotherapy [2,5,20].

In addition to this, metformin inhibits PC growth, which is related to Adenosine Monophosphate-Activated Protein Kinase (AMPK) activation, since AMPK blocks the cell cycle and, consequently, prevents cell growth. AMPK activation by metformin leads to Akt, p70S6 kinase and the Mammalian Target of Rapamycin (mTOR) inactivation, resulting in Akt/mTOR signaling pathway inhibition. mTOR and acetyl-CoA carboxylase (ACC) inhibition blocks protein synthesis and fatty acid synthesis, respectively. Metformin also acts at the level of REDD1 (mTOR and cyclin D1 inhibitor) and inhibits GTPase Rac1, which leads to a reduction in PC metastases; inhibits the epithelial–mesenchymal transformation (EMT) process; decreases the expression c-MYC oncogene, SOX4 transcription factor and FoxM1 transcription factor, the latter two of which are associated with PC cell migration; increases miR30a expression (tumor suppressor); inhibits Pyruvate Kinase M2 (PKM2) phosphorylation and oxidation; and decreases the incidence of prostate intraepithelial lesions [21,22].

Metformin reduces Cycloxygenase-2 (COX-2), Prostaglandin E2 (PGE2) and STAT3 expression, which is an advantage since the COX-2/PGE2/STAT3 axis is responsible for drug resistance, metastasis and tumor relapse. Another mechanism associated with metformin is a reduction in androgen receptor signaling, which may also be related to AMPK activation. The inhibition of IGF-1 by metformin is also essential, since IGF-1 is associated with PC cell growth [21,22].

Additionally, in the group of sulfonylureas, glipizide stands out, because it increases insulin secretion from the pancreatic β cells, which occurs due to the blocking of potassium channels in these cells’ membrane, leading to their depolarization and subsequent opening of the calcium channels, which leads to the entry of calcium ions and insulin exiting [20]. In this way, glipizide inhibits angiogenesis in in vivo models with PC, although it does not block cell proliferation [5,20].

### 2.3. Antiparasitic Drugs

In this group, both anthelmintics and antimalarial drugs seem to be useful in PC treatment. Regarding anthelmintics, mebendazole was originally used to treat parasite infections, but due to its ability to inhibit microtubule polymerization and, consequently, trigger the apoptotic process, it can be used in PC treatment, namely in combination with docetaxel, because of their combined synergistic effect in terms of apoptosis and blocking tumor growth in PC, which has been demonstrated in vitro and in vivo [5,23]. This therapeutic combination shows good synergistic results, since docetaxel binds to tubulin, promotes its polymerization and, consequently, the stabilization of microtubules, instead of mebendazole, which inhibits microtubule polymerization, which affects the dynamics of microtubules and causes aberrant mitoses, namely, problems in achromatic spindle formation, multipolar divisions and aneuploidies [23].

On the other hand, niclosamide decreases the expression of a specific group of androgen receptors, the V7 receptors, which are extremely important in castration-resistant PC, and which are associated with enzalutamide resistance, and for this reason, niclosamide has become useful to sensitize patients to enzalutamide treatment [2,5,24]. In addition, niclosamide acts via the inhibition of signaling pathways that are related to oncogenesis and prostate tumor progression, such as Nuclear Factor Kappa B (NF-κB), Notch, mTORC1, STAT3 and Wnt/β-catenin, where it acts on the LRP6 coreceptor, degrading it [2,12].

Ivermectin, which is also in the anthelmintic group, acts on PC via cell cycle arrest, inducing apoptosis and a decrease in androgen receptor expression, and these are associated with a Proliferating Cell Nuclear Antigen (PCNA) and a decrease in cyclins D and E and an increase in p21. Ivermectin also acts on the FOXA1 protein and Ku70/Ku80 heterodimer, inhibiting them and blocking androgen receptor signaling, E2F1 expression and DNA damage repair, resulting in cell cycle arrest [25,26].

In addition to this, ivermectin causes a synergistic effect in combination with docetaxel, reduces cancer cells’ viability, causing their apoptosis and, consequently, decreases the tumor size. Although the synergic mechanism of these two drugs is still not well understood, it may be related to the inhibition of multidrug resistance (MDR) caused by ivermectin [25].

The literature also presents evidence of antimalarial drug benefits arising from PC treatments such as artemisinin, since both artemisinin and several of its metabolites (dihydroartemisinin) and derivatives (artesunate) induce cell death. Therefore, the anticancer activity of dihydroartemisinin is due to its capacity to inhibit translationally controlled tumor protein (TCTP) expression, while artesunate causes cell cycle arrest, autophagy and angiogenesis inhibition and decreases tumor invasion and metastases and accumulates in lysosomes, promoting ferritin degradation in cancer cells and increasing reactive oxygen species production, which leads to cell death [5,27,28].

In addition to these drugs, chloroquine and its derivative hydroxychloroquine have also demonstrated an ability to interact with nucleotides and induce apoptosis, having shown antitumor, antiproliferative and antimutagenic properties, since they are autophagy inhibitors and, therefore, prevent cancer cells from using autophagy to acquire energy and nutrients [5,17].

Chloroquine and hydroxychloroquine permeabilize the lysosome membrane, resulting in the release of lysosomal enzymes that permeabilize the mitochondrial membrane, producing an apoptosome and, consequently, leading to apoptosis. In the specific case of chloroquine, it induces p53, inhibits anti-apoptotic and survival processes, promotes chromatin folding and, consequently, reduces DNA repair mechanisms [17].

Thus, both chloroquine and hydroxychloroquine seem to be good candidates for administration in radiotherapy (Table 1) and chemotherapy either in neoadjuvant or adjuvant regimens or even concomitantly, but never in monotherapy, because extremely high doses are needed to inhibit tumor growth. Despite this, their synergistic effects would be beneficial because they can act as a chemotherapy in neoplastic cells. However, chloroquine and hydroxychloroquine modulate the patient’s immune response and, for this reason, have the disadvantage of suppressing the immune response against the tumor when administered for long periods, because of their immunosuppressive activity [17]. Quinacrine, another antimalarial drug, also shows favorable results because it activates p53, a tumor suppressor gene [5].

Finally, another antiparasitic drug that can be repurposed is suramin, which reduces serum PSA levels and it has been tested in patients with castration-resistant PC; however, quinacrine’s and suramin’s anticancer mechanisms of action are still unknown [5,29].

### 2.4. Antiarrhythmic Drugs

Digoxin and ouabain are cardiac glycosides in the antiarrhythmic drug class that inhibit the sodium–potassium pump, leading to the intracellular accumulation of calcium and, finally, to PC cell apoptosis, and they also induce cell cycle arrest and autophagy and have antiproliferative properties [2,5,12].

The literature indicates that the sodium–potassium pump has the α and β subunits; the α subunit is downregulated in oncological situations and the glycosylated β subunit is involved in the metastasis process of tumor cells. Thus, these drugs have potential to be repurposed for PC treatment, but more studies are needed to prove their effectiveness, since the existing results are limited due to the small samples used [12].

Thus, digoxin inhibits the topoisomerase II enzyme, produces interleukin 8 and decreases PSA serum levels and tumor growth. This drug also has an inhibitory effect on PC and, when administered in nanomolar concentrations, inhibits HIF-1α and TGFβ, although little is known about this possible candidate [2,5,12]. With regard to ouabain, it sensitizes PC cells to apoptosis, due to the inhibition of survivin, which blocks the apoptotic process; decreases STAT3 expression, which is overexpressed in PC; increases PAR-4 expression; leads to mitochondrial membrane potential loss; and inhibits expression of the HOXB-13 gene, which is overexpressed in the PC and is associated with PC and its metastases, because it is related to the migration and invasion of tumor cells, with ouabain triggering apoptosis via HOXB-13 expression inhibition. Finally, ouabain also inhibits topoisomerase I and II, showing anticancer activity in castration-resistant PC when administered in nanomolar concentrations [2,5,12].

### 2.5. Anti-Inflammatory Drugs

The mechanism of action of non-steroidal anti-inflammatory drugs (NSAIDs) is related to cyclooxygenase (COX) inhibition, which leads to decreased prostaglandin production, although these drugs also act via blocking cell proliferation and inducing apoptosis, and have shown good results in PC treatment, namely in patients who developed resistance to therapy [2,5]. Within the NSAIDs group, selective COX-2 inhibitors stand out, since COX-2 is expressed in PC cells and in the vasculature that feeds them; therefore, drugs such as celecoxib seem to be beneficial, and in addition to this, they also block Akt phosphorylation and activation and inhibit the activity of NF-κB, among other mechanisms that lead to PC cell apoptosis [1,2,5]. On the other hand, the combination of atorvastatin with celecoxib in in vivo studies seems to block tumor growth and tumor progression because of the inhibition of Akt, Erk1/2 and NF-κB, and in vitro, this combination induces autophagy in PC cells [12,30].

However, there are other NSAIDs that are non-selective COX inhibitors and that also have antineoplastic potential, such as indomethacin, which inhibits angiogenesis and the growth of cancer cells through COX-1, COX-2, MAPK and Wnt/β-catenin inhibition and PKC-p38-DRP1-dependent mitochondrial dynamic impairment, and may be effective when used in combination with enzalutamide due to decreased production or utilization of androgens by cancer cells [5]. Diclofenac is another promising drug that inhibits COX, prostaglandin synthesis and the MYC gene family, which is overexpressed in several types of tumors, and seems to interfere with glucose metabolism and its uptake by PC cells, which occurs because the overexpression of MYC is associated with Glucose Transporters-1 (GLUT1) and Lactate Dehydrogenase-A (LDHA) upregulation, culminating in high rates of glucose uptake and glycolysis. By inhibiting MYC through the administration of diclofenac, glucose metabolism is regulated through a decrease in the expression of GLUT1 and LDHA. Diclofenac may also be useful in combination with radiotherapy, because it also has radiosensitizing properties (Table 1) [2].

Similarly to what has already been discussed, acetylsalicylic acid also inhibits COX and prostaglandin synthesis and inhibits NF-κB induced by the Tumor Necrosis Factor (TNF), helping in the overexpression of tumor suppressor genes and in the decrease in the expression of anti-apoptotic genes, making it a candidate for drug repurposing in PC; it is also being evaluated for its efficacy in combination with statins to reduce the levels of the cyclin D1 protein, whose overexpression is associated with tumor development [5]. Furthermore, several studies indicate that NSAIDs can reduce the probability of developing PC through their possible prophylactic effect and also reducing the levels of mortality associated with PC [1,10].

Regarding the steroid anti-inflammatory drug group, also called corticosteroids, dexamethasone stood out in in silico studies for inhibiting the ERG oncogene of PC cells [2,5]. However, currently, dexamethasone is already used in several conventional protocols of chemotherapy and radiotherapy for different tumors types; in the first case, it is administered with the aim of preventing emesis induced by chemotherapy, and in the second case it is given as a prophylactic medicine to treat pain induced by radiotherapy [2,31,32].

### 2.6. Antibacterial Drugs

Monoamine Oxidase-A (MAO-A) inhibitors gained visibility in the topic of repurposing PC drugs after the discovery that high levels of MAO-A were associated with the severity of this cancer, due to the production of reactive oxygen species, and these drugs have demonstrated the ability to reduce metastasis and increase survival in in vivo tests, particularly isoniazid, which is an irreversible MAO-A inhibitor. Therefore, isoniazid may have particular potential in metastatic PC and in more advanced stages in which there is resistance to chemotherapy treatment because MAO-A expression also increases after treatments with docetaxel, resulting in resistance to chemotherapy treatment [33].

Minocycline is a tetracycline derivative that inhibits pro-inflammatory cytokines and metalloproteinases that are associated with tumor invasion and metastasis development, and, for this reason, this drug is in clinical trials to be repurposed for PC treatment, in which it has been found that minocycline inhibits LYN kinase and STAT3, suppresses EMT and consequently blocks metastases [2,34]. On the other hand, the chemical modification of tetracyclines (CMT-3) by activating caspase-3 and caspase-9 and by inhibiting metalloproteinases induces apoptosis in PC cells [2,5].

Through in silico studies, it was concluded that sulfamethoxypyridazine and azlocilline may also be administered in the treatment of PC, because they act in the Fibroblast Growth Factor Receptor (FGFR), which, in the specific case of PC, is associated with angiogenesis and tumor progression; however, there is still a lack of information about the effect of these two drugs in PC treatment [35]. Clofoctol inhibits the translation of proteins from PC cells, inhibiting the growth of the PC itself, which is due to the stress induction in the endoplasmic reticulum and mechanisms of protection against the unfolded protein response (UPR), which suppresses protein translation, and in addition to this, it also inhibits cyclins and promotes cell cycle arrest [2,5]. Finally, nitroxoline inhibits endothelial cell proliferation, limits angiogenesis, induces cell cycle arrest through inhibition of the cyclin D1-Rb-Cdc25A axis and induces apoptosis in tumor cells, because it activates AMPK and inhibits mTOR, which is a fundamental kinase in the cell proliferation process. It further decreases Cdc25A protein expression, which is essential for the proper functioning of the cell cycle, decreases cyclins D1, E, A and B1 levels and inhibits Rb phosphorylation [2,5,36].

### 2.7. Antiviral Drugs

Regarding the antiviral drugs group, nelfinavir proved to be a good candidate for PC treatment, both in monotherapy and in combination with radiotherapy (Table 1) or chemotherapy, since it inhibits regulated intramembrane proteolysis, STAT3 and Akt, induces apoptosis and autophagy, activates both caspase-9 and caspase-6 and inhibits angiogenesis and androgen receptor activation [2,5,15]. So, nelfinavir prevents the cleavage of S2P endopeptidase and promotes SREBP-1 and ATF6 accumulation, which induces endoplasmic reticulum stress and triggers the UPR mechanism, which, after being exceeded, leads to apoptosis [15]. Nelfinavir also inhibits the activation and expression of FASN, which catalyzes the synthesis of long-chain fatty acids and is overexpressed in several tumors. It has been classified as an oncogene, so its inhibition leads to the repression of tumor growth and survival [15].

Saquinavir also demonstrated good results in vitro, both as a monotherapy and in combination with Fluorouracil (5-FU), which may reduce the toxicity associated with chemotherapy [16]. Thus, saquinavir decreases cell proliferation and invasion, has antitumor and anti-angiogenic properties and induces apoptosis, more specifically through the inhibition of the 20s and 26s proteasome and NF-κB and PI3K/Akt inactivation. More than that, saquinavir also showed radiosensitizing properties in PC cells (Table 1), due to its ability to inhibit proteasome function [16].

The drugs didanosine, entecavir, acyclovir, valganciclovir, penciclovir, ganciclovir and valacyclovir have shown in in silico studies that they may be good candidates for PC treatment, as they inhibit the enzyme Poly [ADP-ribose] Polymerase 1 (PARP-1), which plays a fundamental role in the signaling and repair of DNA errors; therefore, by inhibiting PARP in cancer cells, an accumulation of errors that are not repaired and that will lead to cell death can be induced, and PARP is expressed in breast, ovary and prostate tumors [37,38]. On the other hand, glecaprevir and simeprevir are also strong candidates to be repurposed for PC treatment, as they act at GPR 160 and CRHR2, which are part of the G-Protein-Coupled Receptors (GPCRs) that regulate growth and cell metabolism and are involved in the tumorigenesis, angiogenesis and apoptosis of cancer cells. Therefore, certain GPCR members, namely GPR 160 and CRHR2, are overexpressed in several types of cancer, including PC, being promising targets in PC treatment [39].

### 2.8. Antidepressant Drugs

Regarding tricyclic antidepressants, nortriptyline has shown anticancer effects, particularly in PC, because it inhibits cell proliferation and induces apoptosis due to increased intracellular calcium in PC cells and induces cytotoxicity through cell cycle interruption by inhibition of CDK1 and by inhibiting Rb expression, affecting the Rb/E2F complex and, consequently, inhibiting the expression of E2F target genes, since Rb phosphorylation must occur for E2F to be separated from the Rb/E2F complex and activate the expression of genes required for the S-phase transition [40,41,42].

In the group of selective serotonin reuptake inhibitors, sertraline reduces the proliferation of PC cells both in vivo and in vitro, as it also increases calcium intracellular concentration in PC cells and can stop the cell cycle, induces apoptosis through mitochondrial dysfunction and by the production of reactive oxygen species, increasing hydrogen peroxide and lipid peroxidation levels and decreasing glutathione, inhibiting tumorigenesis and angiogenesis and reducing the metastatic potential of PC [6,28]. Sertraline decreases the levels of TCTP, a protein involved in tumorigenesis and cell growth, and in addition to this, it also reduces the expression of Aldehyde Dehydrogenase-1, which is associated with chemotherapy resistance and cancer stem cell formation, CD44, Transcription Factor 8 (TCF8) and Lymphoid Enhancer-Binding Factor 1 (LEF1) [28]. This drug has also been shown to be able to increase the levels of autophagy, cleaved caspase-3 and cleaved PARP-1 and interrupts the viability of PC stem cells through the modulation of iron homeostasis; although this phenomenon is still not well understood, it is known that the imbalance in iron metabolism induces cell death and generates iron-mediated reactive oxygen species [28].

Therefore, cancer stem cells are responsible for tumor progression and resistance to chemotherapy, and it has also been described that stress is associated with the development of PC and that its reduction is important for the success of cancer treatment. So, sertraline was evaluated in PC treatment, and it was noticed that this antidepressant drug decreases the cell viability of PC stem cells, suppresses their growth, inhibits cell proliferation, angiogenesis and tumor cell migration and induces autophagy in cancer stem cells [28].

### 2.9. Antihypertensive Drugs

Propranolol is a β-adrenergic blocker that seems to be advantageous in PC treatment because it interrupts the proliferation of cancer cells, induces apoptosis and inhibits the enzyme Phosphatidic Acid Phosphatase (PAP), which is overexpressed in several types of cancer, and when it is inhibited, there is an accumulation of autophagy markers, as LC3-II and p26. However, these results were obtained after induction with a glucose analogue, 2-Deoxy-D-Glucose (2DG) [5].

Thus, maybe propranolol can be repurposed for neuroendocrine PC treatment, since it decreases neuroendocrine markers, inhibits angiogenesis, and blocks the CREB1-EZH2-TSP1 metabolic pathway. Thus, β-adrenergic blockers are drugs of particular interest to be repurposed for PC treatment because the activation of these receptors is related to angiogenesis and proliferation and migration of tumor cells [12].

On the other hand, prazosin is an antihypertensive drug from the alpha-adrenergic antagonist group that has demonstrated antiproliferative activity by inducing cell cycle arrest via CDK1 inactivation and the apoptosis of PC cells [5]. Prazosin increases phospho-p38α, caspase-8 and caspase-3, reduces S6 kinase and inhibits phosphorylation of PI3K from HER2/Neu, EGF and VEGF, among other mitochondrial apoptosis-inducing factors [5,43]. Among the diuretics, hydroflumethiazide has also shown promising results in silico, although its mechanism of action in PC has not yet been described [35].

Another class of antihypertensives that seems to be advantageous in PC treatment is calcium channel blockers, namely nifedipine, which has been shown in in vivo tests to inhibit the growth of this type of tumor, since it interferes with DNA replication, cell cycle and energy metabolism [41]. Nifedipine also alters the calcium transport that allows the accumulation of intracellular calcium and can lead to apoptosis, and this drug also seems to inhibit angiogenesis and modulates gene expression mediated by androgen receptors, inducing cytotoxicity in cell lines that express these receptors and blocking cell proliferation in PC cells, as it inhibits proteins involved in DNA replication and the cell cycle, such as PCNA, MCM6, MCM3 and MCM4. This drug also affects several metabolic processes, namely the metabolism of alanine, aspartate and glutamate [41,44].

### 2.10. Antifungal Drugs

Terbinafine is an antifungal drug; studies on this drug indicate that it blocks the proliferation of human cancer cells, regulates tumor progression, influences angiogenesis and, when administered via systemic routes, reduces PC mortality by 47% [45]. Furthermore, terbinafine is involved in p53 signaling, triggering apoptosis and can inhibit DNA synthesis to prevent tumor growth and decrease the synthesis of steroids, including cholesterol, which is usually produced in high amounts in cancer cells [45]. However, it is still not known for sure which mechanism of action allows terbinafine to be a good candidate for drug repurposing in PC, as this drug inhibits squalene monooxygenase, which is a fundamental enzyme in cholesterol synthesis [45].

On the other hand, clotrimazole also decreases tumor proliferation and reduces the number of cancer cells in in vitro assays [8]. The mechanisms that are associated with this anticancer activity are its interference in the ionic transport of the plasma membrane, the induction of cell cycle arrest and the inhibition of glycolytic enzymes that are fundamental in the process of tumor growth and are expressed in various types of cancer [46].

In addition, clotrimazole blocks the binding of hexokinase to mitochondria, causing cell death through the release of apoptotic proteins, since this enzyme phosphorylates glucose in the glycolysis process and, when inhibited, prevents glycolysis from occurring, reducing the cellular ATP levels. Therefore, hexokinase is related to tumor growth, being expressed in several tumors, and blocks the cytochrome c release from the mitochondrial outer membrane, preventing apoptosis. Thus, clotrimazole also inactivates Akt, increasing the effect of hexokinase dissociation, affects phosphofructokinase and aldolase, shows an agonist effect on calmodulin and blocks calcium-activated potassium channels, reducing tumor cell proliferation and inducing apoptosis [46].

Itraconazole also inhibits angiogenesis since it binds to VDAC1 and blocks its action in mitochondrial metabolism and inhibits cell proliferation through AMPK activation and mTOR and VEGFR2 inhibition. It also acts on Wnt/β-catenin and, when administered in a dose of 600 mg/day, it has anticancer activity in men with metastatic castration-resistant PC [2,5,47]. Additionally, itraconazole seems to be beneficial both in monotherapy and in combination with chemotherapy drugs, and in this last case, it improves the patient’s survival rate [2]. Furthermore it also reverses P-glycoprotein-mediated resistance to chemotherapy, particularly in the case of docetaxel, where itraconazole resensitizes cells that are resistant to it [2,48].

Finally, ketoconazole is an antifungal that acts in a similar way to abiraterone, an androgen synthesis inhibitor approved for PC treatment that selectively inhibits the enzyme 17α-hydroxylase/C17,20-lyase (CYP17), which is responsible for androgen synthesis and catalyzes the conversion of pregnenolone and progesterone to dehydroepiandrosterone and androstenedione, respectively. Since dehydroepiandrosterone and androstenedione are testosterone precursors, by inhibiting this enzyme, a decrease in circulating testosterone levels can be achieved [9,49,50].

It is also known that 2 h after oral administration of 200 mg of ketoconazole, the serum levels of total and free testosterone are significantly reduced, reaching nadir in a period of 8 h, although this effect is reversible, with testosterone reaching baseline levels within 24 h. However, unlike androgen deprivation therapy, which mainly affects gonadal androgens synthesis, ketoconazole can inhibit the synthesis of gonadal and adrenal androgens, particularly in patients who have variations in the HSD3B1 gene, which is a biomarker of resistance to castration. Another study showed that administration of ketoconazole reduced PSA levels by more than 50% in 20–75% of the included men and increased the time needed for an increase in PSA to occur (3 to 14 months) [50].

Thus, ketoconazole is already considered in some countries a second-line drug for castration-resistant PC treatment; despite not increasing the survival of patients with PC, it was effective as an adjuvant or neoadjuvant to chemotherapy [50]. Therefore, when ketoconazole is administered before docetaxel, there is an improvement in the symptoms of patients with metastatic castration-resistant PC, and it increases their overall survival, although it is expected that patients will respond to docetaxel treatment despite previous treatment with ketoconazole. When ketoconazole is administered after docetaxel in metastatic castration-resistant PC, it is noticed that ketoconazole has benefits, since in a study with 20 participants with metastatic castration-resistant PC with about seven cycles of docetaxel, patients who had adjuvant therapy with ketoconazole revealed a significant 2 months improvement in time to tumor progression [50]. In addition to being used as adjuvant or neoadjuvant therapy, ketoconazole can also be administered in combination with docetaxel, namely 400 mg of ketoconazole twice a day with 55 mg/m^2^ docetaxel for 21 days, which was shown to be reasonably well tolerated by patients in one study, although some experienced neutropenia and tiredness, and significant antitumor activity was found [50].

### 2.11. Immunosuppressant Drugs

The first therapeutic indication of thalidomide was for the treatment of nausea in pregnant women; however, it induced several malformations in the fetus [5,29]. Despite this, it could be repurposed for PC treatment since it has angiogenic activity and PC is known to be highly vascularized compared to the adjacent benign tissue; however, its angiogenic mechanism is still not completely clear, with studies indicating that it may be related to the inhibition of the secretion of angiogenic cytokines, namely, VEGF and FGF, and the inhibition of PI3K/Akt/NF-κB and mTOR [29,51]. Therefore, a study was conducted in which docetaxel was administered in combination with thalidomide, and these patients had less tumor progression, increased survival and improved PSA levels when compared to patients who received docetaxel alone [29].

Leflunomide can also be repurposed, because it inhibits tyrosine kinases and growth factor receptors, such as EGFR, FGFR and PDGFR. The inhibition of PDGFR is of particular interest in the case of PC, since it is expressed in more than 80% of castration-resistant PC patients [2]. This drug inhibits PC cells and, when administered as monotherapy, reduces PSA in more than 50% of men and leads to an improvement in pain, although there are also some studies on combined therapies with leflunomide [2]. Thus, sirolimus, also known as rapamycin, inhibits mTOR and, therefore, blocks cell cycle progression, inhibits the cell growth of castration-resistant PC and lowers PSA levels by about 50%, being another immunosuppressant drug with potential for repurposing for PC [5,52].

### 2.12. Antipsychotic Drugs

In the group of antipsychotics, risperidone stands out for inhibiting 17-β-hydroxysteroid dehydrogenase 10 (17HSD10), which is responsible for the survival of PC cells after treatment with androgen deprivation therapy and overexpressed in PC and in its metastases. It is associated with cell growth and making cells more resistant to oxidative stress, which culminates in tumorigenesis and more aggressive tumor cells, in addition to being a mitochondrial enzyme that catalyzes several metabolic pathways, and its main substrates are estrogens and androgens [2,5,53,54]. On the other hand, in PC, 17HSD10 allows the synthesis of steroids and is also involved in dihydrotestosterone synthesis when in the absence of testosterone, being a useful target in PC treatment [53,54].

Thus, the combination of risperidone with rumenic acid, which was given the designation VAL401, was tested in PC cell lines and showed a significant decrease in cell proliferation, growth and activity, improving patient survival, which us allows to conclude that it is a good candidate for PC treatment. In this orally administered combination, rumenic acid is a functional excipient, as it is essential for risperidone to act on tumor cells [53,54].

Pimozide, which is also in the antipsychotic drugs group, has already been shown to be effective in liver tumor treatment through STAT3 suppression and, for this reason, it was also studied for PC treatment, in which it induced cell cycle arrest and apoptosis, inhibited the migration of PC cells by inhibiting the activation of STAT3 protein, which is involved in cell proliferation, and decreased the expression of cyclin D1, among other mechanisms that allow it to be a promising candidate for PC treatment. It is well tolerated, although it may induce cardiac toxicity; however, it continues to be considered a safe drug [55,56].

### 2.13. Antiepileptic and Anticonvulsant Drugs

Valproic acid inhibits Class I Histone Deacetylases (HDAC I), and the DNA region that contains the main anticancer genes is associated with these enzymes that, when inhibited by valproic acid, stop to silence these genes. Valproic acid administration for long periods decreases PC proliferation, increases both caspase-2 and caspase-3 activity [2,5], decreases the inflammatory process associated with the tumor, the proliferation of cancer cells and angiogenesis, increases the expression of miR-34a, which inhibits the migration of PC cells, and stimulates the metastasis suppressor protein NDRG1 [2]. However, acute treatments with valproic acid resulted in increased expression of androgen receptors and increased levels of PSA, and it is possible to reverse this effect with prolonged administration of valproic acid [2]. Therefore, valproic acid has shown good results when administered in prolonged monotherapy, but has shown even more promising results when administered in combination with cytotoxic drugs such as 5-FU, epirubicin, cyclophosphamide and doxorubicin [35]. Valproic acid also showed good results with metformin and simvastatin, two drugs with potential to be repurposed for PC treatment and which were previously discussed in this work, since with metformin, they induce synergistic apoptosis and strongly inhibit the proliferation of PC cells [35] and with simvastatin, lead to the sensitization of PC cells to docetaxel treatment, reducing the phenomenon of resistance through the inhibition of YAP, which functions as a transcription regulator and is associated with PC [5].

Regarding levetiracetam, it acts on the SV2A protein, which is overexpressed in neuroendocrine PC and mast cells that infiltrate prostate adenocarcinoma and, for this reason, in in vitro and in vivo studies, levetiracetam inhibited the proliferation of neuroendocrine PC cells and mast cell degranulation. Thus, levetiracetam may inhibit the development of neuroendocrine PC after androgen deprivation therapy and block the progression of adenocarcinoma through the inhibition of mast cells. On the other hand, like valproic acid, it also inhibits HDAC, but this study demonstrated that extremely high doses of levetiracetam are required to cause these effects [57].

### 2.14. Bisphosphonates

Zoledronic acid belongs to the bisphosphonate group and is administered for osteoporosis because it prevents and treats bone lesions, and because it inhibits osteoclast activity in bones [2,5,13,58]. Due to its action at the bone level, it has also been used in bone metastases treatment, namely in metastatic PC [59] and, in addition to this, it has been shown to have anticancer mechanisms in PC, namely cell cycle arrest, inhibition of tumor cell proliferation and viability and angiogenesis, induction of apoptosis associated with the mevalonate pathway and both caspase-3 and caspase-9 activation and changes in metalloproteinase expression [2,5,60]. Zoledronic acid also inhibits the migration of tumor cells and prevents the formation of PC micrometastases by inhibiting RANKL/RANK. Therefore, zoledronic acid increases the levels of reactive oxygen species, the expression of the pro-apoptotic protein Bax and the permeability of the cell membrane and decreases the expression of the anti-apoptotic protein Bcl-2, triggering apoptosis in PC cells. In addition to these mechanisms, zoledronic acid also acts in the Ras/Erk1/2 pathway, since it inhibits Ras protein, which is associated with the survival of tumor cells and, consequently, Erk 1/2, Akt and HIF-1α; reduces glycolysis; inhibits Bcl-2 and Bad phosphorylation; and inhibits P-glycoprotein, STAT3, VEGF, FGF-2 and PDGF-BB [60].

This bisphosphonate also has benefits when administered in combination with other drugs, namely docetaxel, with which it increased survival in men with castration-resistant PC and those with celecoxib, although in this case it did not increase survival [2,5]. Therefore, due to all these properties, zoledronic acid is already used in some countries as a repurposed drug for PC treatment [2].

### 2.15. Medication for Alcoholism

Disulfiram is a P-glycoprotein inhibitor capable of inhibiting chemotherapy efflux, and P-glycoprotein is overexpressed in cancer cells, leading to resistance to certain treatments [61]. So, when disulfiram is administered in combination with doxorubicin, an increase in the cytotoxicity of the chemotherapy drug is observed [61]. On the other hand, disulfiram inhibits 5-methyl cytosine (5meC) and aldehyde dehydrogenase and, consequently, leads to the accumulation of acetaldehyde in the blood and inhibits formaldehyde oxidation in cancer cells, leading to apoptosis [5]. Furthermore, it can also restore tumor suppressor genes, because it acts as an inhibitor of DNA methyltransferases (DNMT1), decreases APC and RARB methylation, induces metallothionein expression and DNA demethylation in PC cells and reduces tumor growth in vivo [2,5].

### 2.16. Other Drugs

Ormeloxifene is an estrogen receptor modulator (Table 2) and has been proven to be a good candidate for drug repurposing in several cancer types, namely PC, in which it blocks the invasion of cancer cells, prevents metastasis, induces apoptosis and delays tumor growth, which may be related to its ability to inhibit the oncogenic signaling of β-catenin, and consequently, inhibit the EMT [2,5]. This drug also activates GSK3β, inhibits TCF-4, Mcl-1, cyclin D1 and CDK4 and induces p21 and p27, with studies indicating that it can be used in PC treatment both in monotherapy and in combination with other drugs [62].

Mifepristone is a steroid with antiprogesterone action approved for pregnancy termination (Table 2); however, it has been shown to be useful in the treatment of hormone-sensitive PC and castration-resistant PC due to the induction of pro-apoptotic transcription factors, inhibition of tumor growth and blockage of overexpressed receptors in PC cells, such as progesterone, estrogen and glucocorticoid receptors. Thus, the combined administration of mifepristone with other drugs, such as corticosteroids, ketoconazole or 5-α reductase inhibitors, prevents the increase in androgen levels in patients with PC [2,5].

Cinacalcet is used in the treatment of secondary hyperparathyroidism, and it has shown that when administered in combination with irinotecan, it can obtain good results in PC treatment, which is due to the action of cinacalcet on GPR 160 and CRHR2 and the action of irinotecan on GPR 160 [39].

Amlexanox has a therapeutic indication for the treatment of ulcers, allergic rhinitis and asthma and has been involved in trials analyzing its potential for PC treatment because it is an inhibitor of the IKBKE/TBK1 kinases that stop cell cycle and induce apoptosis. After the inhibition of IKBKE, there was a decrease in c-MYC protein levels and, consequently, a decrease in the binding of c-MYC to androgen receptors, and because c-MYC is regulated by YAP via the inhibition of IKBKE, YAP levels also decreased; however, contradictorily, the investigators detected that the inhibition of IKBKE increased the expression of YAP mRNA, which led to the deduction that the decrease in protein levels was due to a YAP turnover. It was also verified that IKBKE is related to androgen receptors, since after the inhibition of IKBKE there was a decrease in gene expression regulated by androgen receptors, which culminated in the inhibition of proliferation, migration and capacity for colony formation of PC cells and in reducing tumor size and increasing survival [63].

Naftopidil, a selective adrenoreceptor A1D antagonist administered in urinary symptom treatment associated with benign prostatic hyperplasia, improved the results of radiotherapy sessions (Table 1) in in vitro tests [5] conducted to verify PC cell growth inhibition and Akt phosphorylation inhibition [2]. On the other hand, naftopidil, when administered for periods longer than 3 months, reduced the incidence of PC, reduced the levels of Bcl2 which is a marker of resistance to apoptosis, inhibited the phosphorylation of activated Smad2, induced apoptosis and showed cytotoxicity in PC cell lines [43].

Finally, memantine is used in Alzheimer’s disease treatment; however, it induces apoptosis in PC cells through an increase in caspase-3 and caspase-9 levels and the expression of the pro-apoptotic protein Bax and CDK4, decreases CCND1 and decreases the expression of the c-MYC protein, Bcl-2, survivin and HIF-1α and inhibits cell cycle progression [64]. In addition, memantine is an NMDA receptor antagonist, like ifenprodil, which also shows promising results, and NMDA receptors seem to be a useful target in the treatment of various malignancies because their blockade increases survival in patients with metastatic tumors. Thus, NMDA receptor antagonists, namely memantine and ifenprodil, inhibit PC cell proliferation [35].

## 3. Conclusions

There are several drugs whose original therapeutic indication is not anticancer, but which have proven in in vitro, in vivo or in silico studies that they are potentially useful and effective drugs for PC treatment either in monotherapy or in combination with antineoplastic drugs, such as chemotherapy. Therefore, several action mechanisms were identified that explain the anticancer properties of these drugs and that make them promising candidates for PC treatment. In summary, repurposed drugs have proved to be an effective strategy in PC, because several candidates were found with characteristics that will save time and money in the investigation of this cancer treatment.

## Figures and Tables

**Table 2:** Examples of drugs that were originally used in women but show anticancer activity in prostate cancer.

Drug Class	Drug	Original Use	Anticancer Mechanism	References
Selective estrogen receptor modulator	Ormeloxifene	Oral contraceptive	Inhibit EMT by repressing N-cadherin and β-catenin	[2,5]
Antiprogesterone	Mifepristone	Pregnancy termination	Suppression via blocking cell surface progesterone receptors; induction of TFG-β-1	

## Data Availability

Not applicable.
